# Identification of a rare [^G^*γ*(^A^*γδβ*)^0^] -thalassemia using tandem mass spectrometry

**DOI:** 10.1002/pmic.202300495

**Published:** 2024-01-11

**Authors:** Anikha Bellad, Kannan Rangiah, Gajanan Sathe, Gourav Dey, Pragalatha Kumar Appadorai, Hemalatha Lokanatha, Pradeep Rudra Murthy, Aruna Gowdra, Akhilesh Pandey

**Affiliations:** 1 Manipal Academy of Higher Education, Manipal, Karnataka, India; 2 Institute of Bioinformatics, International Technology Park, Bangalore, Karnataka, India; 3 Department of Pediatrics, Indira Gandhi Institute of Child Health, Bangalore, Karnataka, India; 4 Department of Pathology, Indira Gandhi Institute of Child Health, Bangalore, Karnataka, India; 5 Department of Biochemistry, Indira Gandhi Institute of Child Health, Bangalore, Karnataka, India; 6 Department of Laboratory Medicine and Pathology, Mayo Clinic, Rochester, Minnesota, USA; 7 Center for Individualized Medicine, Mayo Clinic, Rochester, Minnesota, USA

**Keywords:** globin, hemoglobinopathies, HPLC, LC-MS/MS, *δβ*-thalassemia

## Abstract

Thalassemias are a group of inherited monogenic disorders characterized by defects in the synthesis of one or more of the globin chain subunits of the hemoglobin tetramer. Delta-beta (*δβ*-) thalassemia has large deletions in the *β* globin gene cluster involving *δ*- and *β*-globin genes, leading to absent or reduced synthesis of both *δ*- and *β*-globin chains. Here, we used direct globin-chain analysis using tandem mass spectrometry for the diagnosis of *δβ*-thalassemia. Two cases from unrelated families were recruited for the study based on clinical and hematological evaluation. Peptides obtained after trypsin digestion of proteins extracted from red blood cell pellets from two affected individuals and their parents were analyzed using liquid chromatography-tandem mass spectrometry (LC-MS/MS). Mass spectrometric analysis revealed a severe reduction in *δ, β*, and A*γ* globin proteins with increased ^G^*γ* globin protein in the affected individuals. The diagnosis of ^G^*γ*(^A^*γδβ*)^0^ -thalassemia in the homozygous state in the affected individuals and in the heterozygous state in the parents was made from our results. The diagnosis was confirmed at the genetic level using multiplex ligation-dependent probe amplification (MLPA). Our findings demonstrate the utility of direct globin protein quantitation using LC-MS/MS to quantify individual globin proteins reflecting changes in globin production. This approach can be utilized for accurate and timely diagnosis of hemoglobinopathies, including rare variants, where existing diagnostic methods provide inconclusive results.

## 1 Introduction

Thalassemias are among the most common monogenic disorders worldwide. They are classified based on the globin gene involved. The most commonly seen thalassemias are *α*- and *β*-thalassemias, which are caused by reduced or absent production of *α*- or *β* globin chains. Other rare forms are due to abnormalities in other globin genes including *δ, γ, ε* chains. According to the World Health Organization (WHO) update on beta thalassemia, India accounts for a carrier frequency of 3%–4%, which corresponds to ~35–47 million carriers for the disease [1, 2]. The majority of mutations which affect the expression of the *β*-globin gene are point mutations. However, larger deletions or rearrangements of DNA have also been linked to altered gene expression in the *β*-globin cluster. An example of a type of thalassemia associated with large deletions is *δβ*-thalassemia. It is a rare form of thalassemia that is characterized by decreased or absent production of both *δ*- and *β*-globin chains with elevated levels of fetal hemoglobin (HbF) beyond infancy [3].

**Figure 1:**
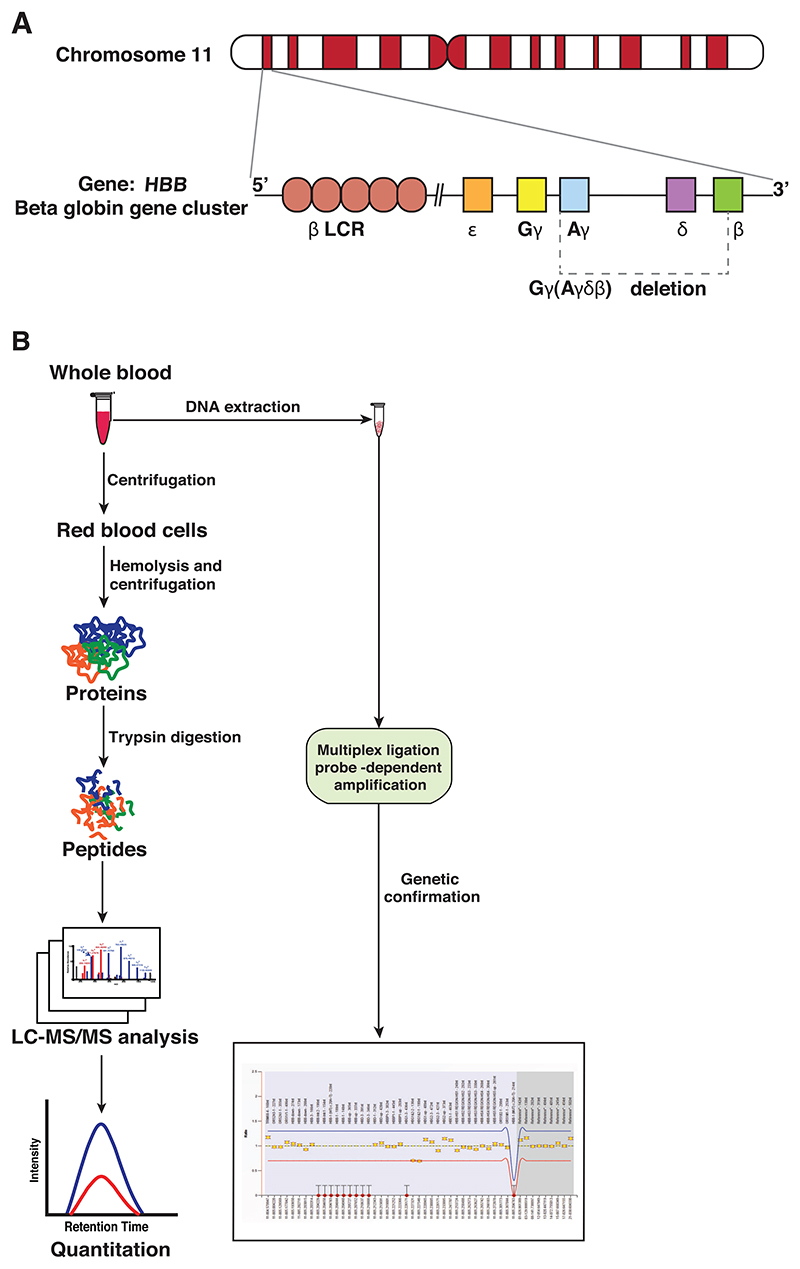
A tandem mass spectrometry-based strategy to investigate G*γ*(A*γδβ*)^0^ deletion. The organization of *β*-globin gene cluster on chromosome 11 is shown in (A). The human *β*-globin gene cluster consists of five genes arranged on chromosome 11. The genes are indicated by boxes and are also in the order of their expression during development: 5′-ε-, G*γ* -, A*γ*-, *δ*-, and *β*-globin gene. The *β*–locus control region (*β*–LCR), a major regulatory element located upstream of the genes of the cluster, is depicted in the figure. The region of the deleted gene cluster in the present study is indicated by a dashed line in the figure. Overview of the quantitative proteomic analysis of globin proteins in cases of *δβ*-thalassemia is shown in (B). Whole blood samples obtained from probands and their parents were lysed and proteins were extracted. The extracted proteins were digested with trypsin and the peptides analyzed on an Orbitrap Fusion tribrid mass spectrometer followed by data analysis. The DNA extracted from the samples was subjected to multiplex ligation-dependent probe amplification (MLPA) analysis.

*δβ*-thalassemia mutations have been reported in various ethnic groups across the globe. They include several deletion mutations, including Indian, Turkish, German, Japanese, Black, Sicilian, Thai, and Spanish types [4, 5]. Although the exact prevalence of this condition is not known, it is highly variable across different population groups. Based on a study from Brazil [6] and Thailand [7], the prevalence of these abnormalities ranges from < 1:10,000 in Brazil to as high as 4% in the Thai population. Very few cases have been reported from different regions of India [8–10]. According to one multicentric study from India, the prevalence rate of *δβ*-thalassemia-trait in different cities in India ranges from 0.02% to 0.73% [11].

(*δβ*)^0^-thalassemia is classified into two molecular subtypes: ^G^*γ*^A^*γ*(*δβ*)^0^ and ^G^*γ*(^A^*γδβ*)^0^-thalassemia on the basis of ^G^*γ*- and ^A^*γ*-globin production, along with *δ* and *β*-globin chains. While both ^G^*γ*- and ^A^*γ*-globin chains are synthesized in the former, only ^G^*γ*-chains are seen in the latter [12]. The organization of the *β*-globin gene cluster on chromosome 11 along with deletions reported in the current cases is shown in Figure 1A. Although a distinction between these two subtypes can be made using genetic testing, high-performance liquid chromatography (HPLC) or capillary electrophoresis cannot distinguish between these different molecular subtypes of *δβ*-thalassemia. Most functionally characterized ^G^*γ*(^A^*γδβ*)^0^-thalassemias are heterozygous deletions, with limited reports of homozygous deletions that represent simple chromosomal rearrangements in the *β*-globin gene cluster [10, 13].

The clinical phenotype of *δβ*-thalassemia is highly variable. Patients often present as thalassemia intermedia, the homozygous mutation in *δβ*-thalassemia presents with variable severity ranging from mild anemia to thalassemia major [14–17]. Owing to the absence of *δ* and *β*-globin chain production in homozygotes, they cannot synthesize HbA2 and HbA; and HbF comprises ~100% of the hemoglobin produced [18, 19], whereas heterozygotes for *δβ*-thalassemia exhibit a modest elevation of HbF (5%–20%) with hypochromic microcytic blood picture on peripheral smear [15]. Individuals with heterozygous *δβ*-thalassemia remain clinically unaffected with red blood cell changes typical of *β* thalassaemia heterozygotes, but with normal levels of HbA2 [20]. This finding can also be seen in another hematological disorder namely hereditary persistence of fetal hemoglobin (HPFH). The main difference between these two conditions can be confirmed by alpha-beta-globin chain imbalance and/or DNA analysis. However, the analysis of globin chain imbalance is not utilized for routine screening [21].

Detection of large deletions within the *β*-globin gene cluster is important because of its significance in evaluation of unresolved thalassemia-related cases. The homozygosity or compound heterozygosity of these mutations with *β* thalassemia can result in severe disease which can significantly alter the treatment strategy [22, 23]. Molecular characterization of thalassemia deletions is important not only for diagnosis and treatment of carriers but also for genetic counselling to offer prenatal diagnosis to offer women the option of preventing the birth of the affected child.

Currently, HbA2 determination by HPLC or capillary electrophoresis plays a key role in screening for *β*-thalassemia, and an increase in the HbA2 fraction is used as the marker to diagnose the *β*-thalassemia trait. However, co-inheritance of *β*- and *δ*-thalassemia or *β*-thalassemia trait with normal HbA2 can compromise the diagnosis of *β*-thalassemia carriers when based on the elevated HbA2 level alone. On the other hand, commonly used genetic diagnostic tests target point mutations and small insertions/deletions and tend to miss large deletion mutations. Thus, a confirmatory diagnosis requires other molecular assays such as Southern blotting, fluorescence in-situ hybridization, quantitative polymerase chain reaction, multiplex ligation-dependent probe amplification and/or gap-PCR [23–26] to detect large deletions. These limitations in existing assays are the sources of diagnostic pitfalls in carrier screening and genetic counselling. Thus, it is crucial to have efficient tools to cover both small and large deletions in order to give the patients the most appropriate genetic counselling [27].

In the current study, we report two cases of homozygous ^G^*γ*(^A^*γδβ*)^0^-thalassemia associated with a gross deletion in the *β*-globin gene cluster in two unrelated Indian families, both of which were identified using high-resolution mass spectrometry.

## 2 Materials and Methods

### 2.1 Recruitment of cases

Clinically suspected cases of *δβ*−thalassemia from two unrelated families were recruited for the study based on routine hematological and biochemical investigations from the Indira Gandhi Institute of Child Health, Bangalore, India. Whole blood EDTA samples were collected from both the probands, and their parents with prior written informed consent for performing hematological and molecular investigations. A proband is a person who serves as the starting point for the genetic study of the family. The study was approved by the Institutional Ethics Committee at Indira Gandhi Institute of Child Health. All experiments were performed in accordance with the Declaration of Helsinki; the ICMR National Ethical Guidelines for Biomedical and Health Research involving Human Participants 2017 and other relevant guidelines and regulations.

### 2.2 Hematological and biochemical investigations

The complete blood count and red blood cell indices were measured from freshly collected whole blood samples using an automated cell analyzer. Hematoxylin and eosin-stained peripheral blood smears were examined for blood cell morphology and for quantitative assessment of the hemoglobin fractions, HbF, HbA, and HbA2 analysis was performed by cation-exchange HPLC.

### 2.3 Sample preparation for LC-MS/MS analysis

Whole blood samples obtained from patients and their parents were centrifuged at 3000 rpm for 10 min and the plasma was separated. The packed cell pellets were then washed using normal saline three times before lysis with five volumes of ice-cold distilled water. The hemolysate was centrifuged at 20,000 x *g* for 10 min at 4°C to remove the erythrocyte membranes, and the supernatant was used for subsequent analysis. Protein estimation of the samples was performed using the bicinchonic acid (BCA) assay.

### 2.4 In-solution digestion

An equal amount of protein (100 *μ*g) from each sample was subjected to reduction using 10 mM dithiothreitol (pH 8.0 at 60°C for 30 min.) followed by alkylation using 20 mM iodoacetamide (at room temperature, in the dark for 20 min.). The samples were then digested by trypsin (1:20, Promega) at 37°C overnight. The tryptic peptides obtained were purified using a Sep-Pak C_18_ Cartridge (Waters). The desalted peptides were vacuum-dried and stored at −80°C until LC-MS/MS analysis.

### 2.5 LC-MS/MS analysis

The vacuum-dried peptides were reconstituted in 0.1% formic acid and analyzed on an Orbitrap Fusion Tribrid mass spectrometer (Thermo Scientific) interfaced with an Easy-nLC II nanoflow liquid chromatography system (Thermo Scientific). The peptides (1 *μ*g) were loaded onto a trapping column (75 *μ*m x 2 cm, Acclaim PepMap) at a flow rate of 3 *μ*L/min and were separated on an analytical column (75 *μ*m × 50 cm, Acclaim PepMap RSLC C_18_) at a flow rate of 280 nL/min by using a step gradient of 5−30% solvent B (95% acetonitrile in 0.1% formic acid) over 70 min. The total run time was 120 min. A survey full-scan MS (from m/z 350 to 1600) was acquired in time-dependent acquisition mode in the Orbitrap with a resolution of 120,000 at 400 m/z. The automatic gain control (AGC) target for MS1 was set to 4 × 10^5^ with an ion injection time of 50 ms and dynamic exclusion of 30 s. The most intense ions with charge states 2−6 were isolated in 3-s cycle. Peptides were then fragmented by higher energy collisional dissociation (HCD) with 32% normalized collision energy. Fragment ions were acquired over an m/z 110−2000 scan range with the AGC target for MS/MS set at 1 × 10^5^ with an ion injection time of 100 ms. Resolution for MS/MS was set to 15,000 at 400 m/z. with a 10 ppm mass window. To evaluate the reproducibility of our analytical pipeline, we quantitatively compared the samples run in triplicate. The variability in globin protein quantitation was expressed as coefficient of variation (CV%). The schematic workflow of the experiment is summarized in Figure 1B.

### 2.6 Quantitative proteomics analysis

The acquired mass spectrometric data was searched against the Human RefSeq protein database (Version 108, containing protein entries with common contaminants) using SEQUEST HT and MAS-COT search algorithms through the Proteome Discoverer (PD) platform (version 2.4, Thermo Scientific). The search parameters included trypsin as the protease with a maximum of two missed cleavages, carbamidomethylation of cysteine as a fixed modification with oxidation of methionine and acetylation at protein N-terminus as variable modifications. MS and MS/MS mass tolerances were set to 10 ppm and 0.05 Da, respectively. A False Discovery Rate (FDR) of 1% was set at both the peptide-spectrum match (PSM) and protein levels. The Minora node was used for label free quantitation (LFQ) of the identified globin proteins. The relative abundance of *α, β, δ*, ^G^*γ*, and ^A^*γ*-globin chains was represented using Graph Pad Prism version 5.0 (GraphPad Software Inc., San Diego, CA, USA). The globin chain ratios were calculated to detect any imbalance in globin chains among homozygous proband and heterozygous parents.

### 2.7 Calculation of *β*:*α* and ^G^*γ*:^A^*γ* globin ratios

The unique peptides from *α, β*, ^G^*γ*, and ^A^*γ*-globin proteins were used to calculate the globin ratios. The reproducibility of the globin ratios were expressed as CV%. The relative abundances of the respective peptides were determined using the quantitation values based on their peak intensity directly obtained using PD version 2.4 software. The summed abundances of the respective unique peptides were used to calculate the *β*:*α* and ^G^*γ*:^A^*γ* globin ratios.

## 3 Molecular Analysis

MLPA analysis was carried out for genomic analysis of the samples. DNA was extracted from peripheral blood of probands and their parents using a QIAamp DNA minikit (Qiagen) following the manufacturer’s protocol. MLPA was performed using the SALSA MLPA probe mix (P102) HBB (MRC Holland, Amsterdam, Netherlands) according to the manufacturer’s protocol. This mix contains 49 MLPA probes with amplification products between 130 and 502 nucleotides. These included 40 probes for the beta-globin gene cluster and its flanking regions, in addition to nine reference probes that detect autosomal chromosomal locations. Approximately 200 ng of DNA was used for the assay. Ligation and amplification were carried out on a thermal cycler and the amplified products were separated by capillary electrophoresis. The data was analyzed using Coffalyser software from MRC-Holland. In addition to this, *HBB* gene mutation analysis, based on Sanger sequencing was performed in both parents in one of the families (Family 2) to exclude the possibility of homozygous *β*-thalassemia or double heterozygosity for *β*-thalassemia and *δβ*-thalassemia.

## 4 Results

### 4.1 Case description

#### Case 1

**Figure 2:**
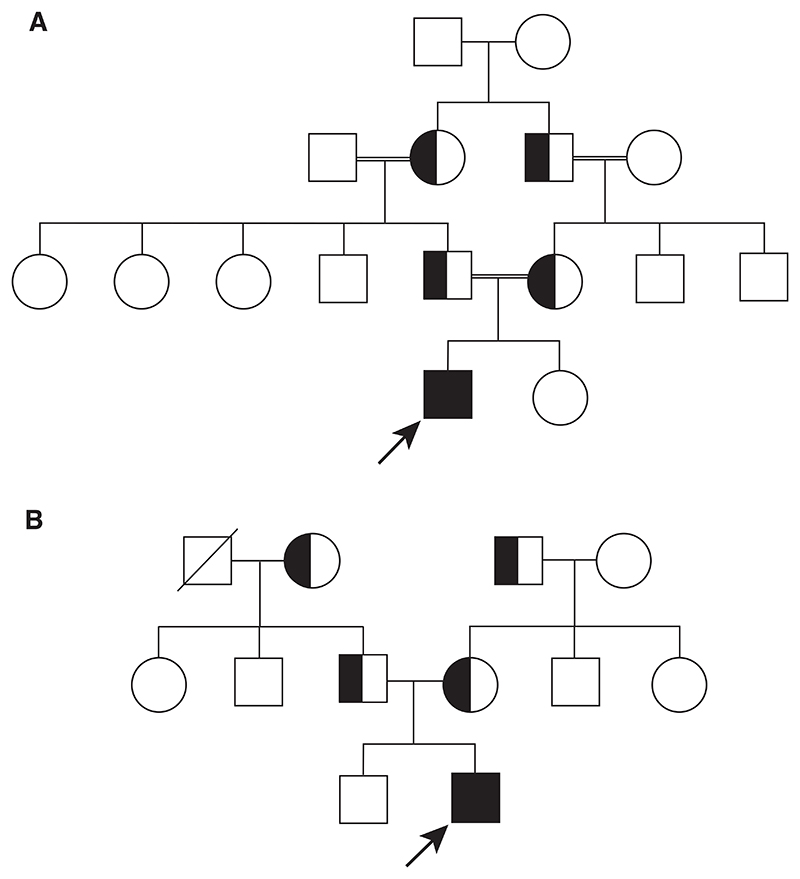
Pedigree analysis of the families with affected individuals. A pedigree analysis indicating consanguinity is shown for Family 1 (A) and Family 2 (B). The arrows denote the probands.

A 10-year-old male child, 1st born to a multigenerational consanguineous family (Figure 2A) presented to the outpatient department of Indira Gandhi Institute of Child Health with a history of weakness, recurrent upper respiratory tract infection and fever with poor scholastic performance. He was on infrequent blood transfusions with multi organ involvement. Hematological investigations revealed severe anemia with abnormal red cell distribution showing dimorphic population of RBCs. In addition, hemoglobin variant analysis by HPLC revealed an extremely elevated HbF level (96.6%) with very low HbA (3.4%) and absent HbA2 peak (0%) (Figure S1A). This patient had a younger sibling who was found to be in good general health with normal development. However, she was not available for further evaluation. Family studies of both parents revealed mild anemia with abnormal hemoglobin pattern on HPLC analysis with elevated levels of HbF with normal HbA2 levels.

#### Case 2

An 8-month-old male child, 2nd born out of a nonconsanguineous marriage (Figure 2B) was brought to the outpatient department of Indira Gandhi Institute of Child Health by his mother. He had a history of failure to thrive and mass in the left side of upper abdomen. The routine hematological investigations of the child revealed mild anemia with the peripheral blood smear finding of hemolytic anemia. Based on a suspicion of the presence of hemoglobin variant, Hb pattern analysis by HPLC was performed which revealed complete replacement of HbA and HbA_2_ by HbF (Figure S1B). Family studies of Hb pattern analysis by HPLC on both parents revealed increased levels of HbF with reduced HbA2.

Because of a peripheral blood smear that demonstrated hemolytic anemia and the HPLC findings, a provisional diagnosis of homozygous *δβ*-thalassemia in both of the probands and a heterozygous condition in parents was made. However, confirmation by genetic analysis was suggested. We therefore performed direct globin quantitation in these cases to demonstrate the utility of tandem mass spectrometry in the definitive diagnosis of large beta-globin deletions such as *δβ*-thalassemia. The summary of the hematological and biochemical investigations in the families is provided in Table 1.

### 4.2 Globin chain quantitation and globin chain imbalance

**Figure 3:**
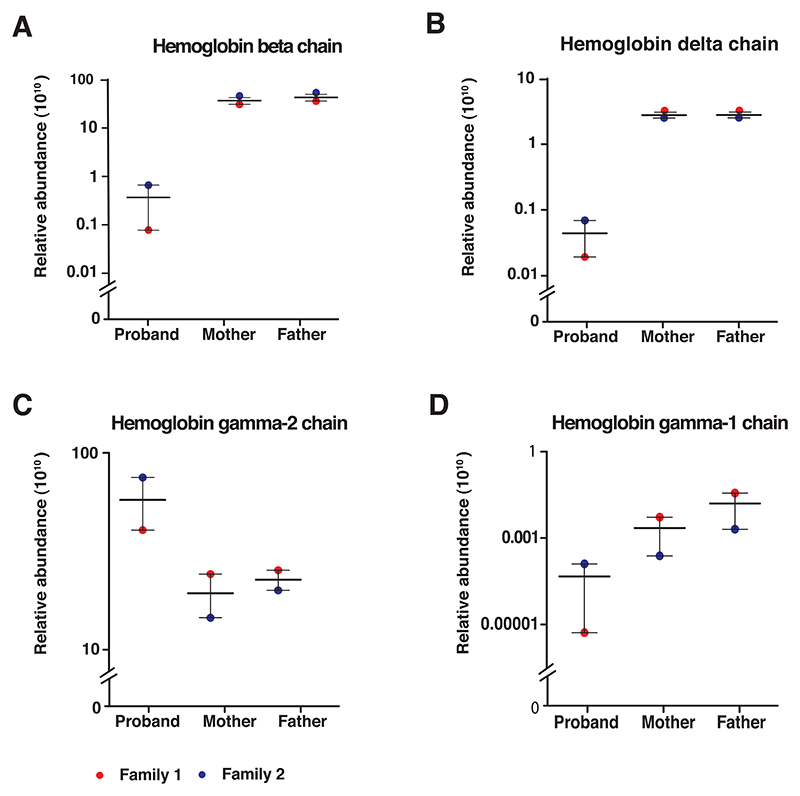
Relative levels of globin chains from homozygous probands and heterozygous parents. The panels show the relative abundance levels of various globin chains as indicated. (A) HBB, (B) HBD, (C) HBG1 (A*γ*), and (D) HBG2 (G*γ*). The means are indicated with a horizontal line and along with the lowest and highest values marked as red and blue filled circles, to indicate the range. The figure depicts severe reduction in HBB, HBD and HBG1 globin proteins with high levels of HBG2 in the homozygous proband as compared to heterozygous parents.

In the present study, we report the direct quantitation of globin chains in suspected cases of *δβ*-thalassemia using an LC-MS/MS-based approach. We identified different globin chains from homozygous probands and heterozygous parents of both families and quantified them using a mass spectrometry-based label-free quantitation. From the identified globin proteins, *β, δ*, and A*γ* globin proteins were significantly altered in the homozygous probands compared to heterozygous parents. Our findings revealed that proband 1 had a severe reduction in *β*- and ^A^*γ*-globin chains by > 400-fold and 1000-fold, respectively, as compared to his heterozygous parents. Similarly, proband 2 had > 75-fold decrease in *β*-globin chains compared to his heterozygous parents. Furthermore, a similar reduction was observed in *δ* and ^A^*γ* globin chains in the two probands compared to their heterozygous parents. There was also an observed increase in ^G^*γ*-globin chains in the probands, by— > 1.5-fold and > 4-fold in proband 1 and proband 2, respectively, compared to their heterozygous parents. A comparison of relative abundances of the globin chains in homozygous probands and heterozygous parents is shown in Figure 3A–D.

**Figure 4:**
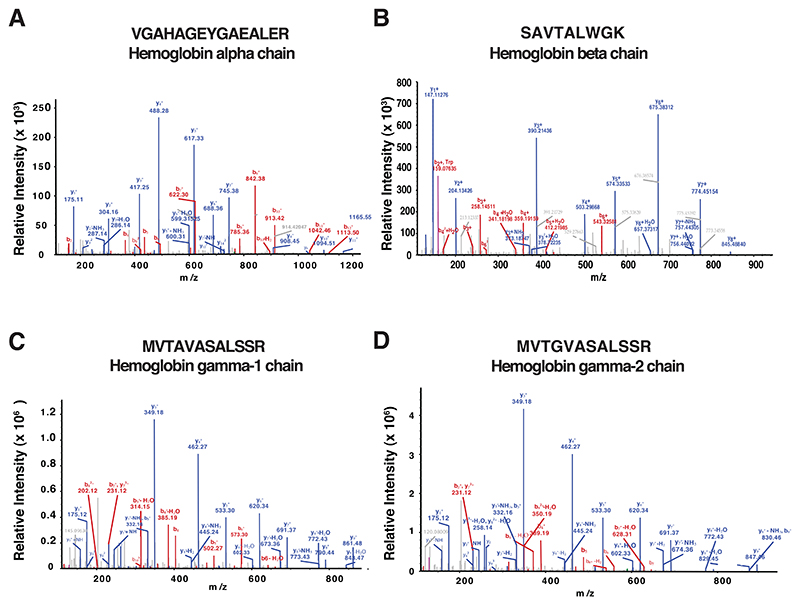
Representative MS/MS spectra of the peptides used for globin ratio quantitation. (A) Hemoglobin alpha chain (HBA). (B) Hemoglobin beta chain (HBB). (C) Hemoglobin gamma-1 chain (HBG1). (D) Hemoglobin gamma-2 chain (HBG2). The panels depict annotated fragment ion spectra of the indicated peptides with the b and y series of ions marked in red and blue, respectively.

We next measured the globin imbalance based on the *β*:*α*globin and ^G^*γ*:^A^*γ* globin ratios. These ratios would aid in differentiating *δβ*-thalassemia from a closely related condition, HPFH. The ^G^*γ*:^A^*γ* ratio would also help in determining the molecular subtype of the *δβ*-thalassemia. We used unique peptides generated by tryptic digestion of each globin protein to calculate these ratios. The three unique peptides from *α*- and *β*-globin, and one each from ^G^*γ*- and ^A^*γ*-globin were selected for globin ratio analysis (Table 2). The summed abundances of these unique peptides was used to calculate *β*/*α* and ^G^*γ*/^A^*γ* globin ratios. The representative MS/MS spectra of the peptide sequences used in the globin-chain ratio calculation are shown in Figure 4A–D.

**Figure 5:**
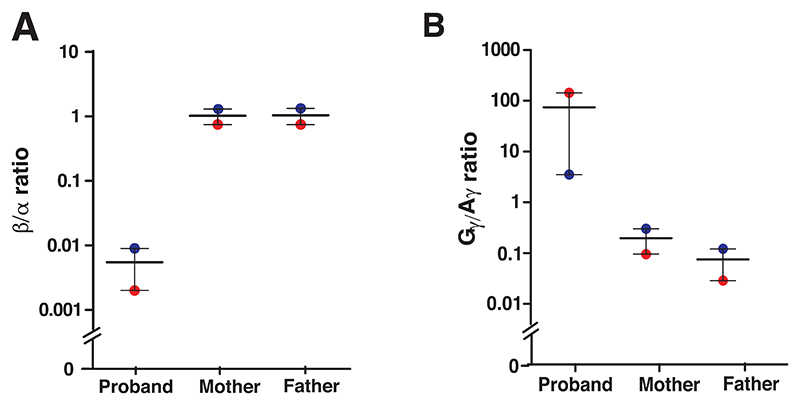
Globin abundance ratios in homozygous proband and heterozygous parents from two families. (A) Beta/alpha (*β*/*α*) globin ratio: The figure depicts reduced *β*/*α* globin ratio in the proband compared to parents suggesting the high degree of beta to alpha globin imbalance in the homozygous *δβ*-thalassemia. (B) G-gamma/A-gamma (G*γ*/A*γ*) globin ratio: The figure depicts very high G*γ*/A*γ* globin ratio in probands of two families compared to parents suggesting the suppression of A*γ* production in the homozygous G*γ*(A*γδβ*)^0^-thalassemia. The means with range are plotted in the figure.

The *β*:*α* globin chain ratio showed a drastic reduction in the homozygous probands compared to their heterozygous parents (Table 3; Figure 5A). Also, the severe reduction in ^A^*γ*-globin in the probands led to unusually high ^G^*γ*/^A^*γ* globin ratios with varied range of ratios among heterozygous parents (Table 3; Figure 5B). Our findings indicate a significant reduction in the synthesis of ^A^*γ, δ*, and *β*-globin chains with intact ^G^*γ*-globin chain in the probands and partial reduction of these proteins in their parents.

## 5 Method Performance

**Figure 6:**
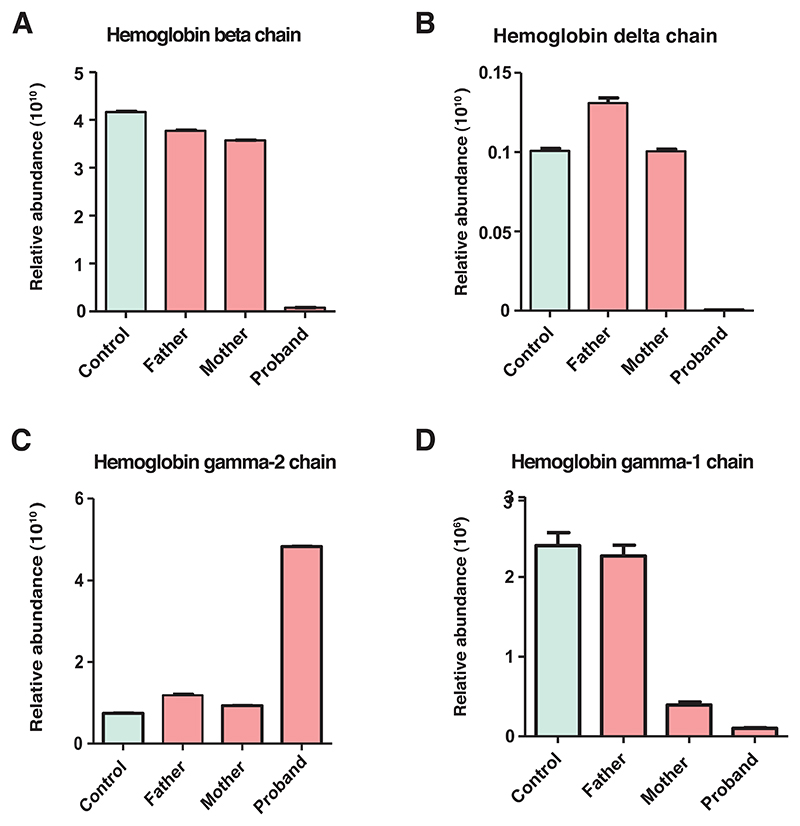
Bar charts showing the coefficient of variation for intra-assay measurements of hemoglobin chain by tandem mass spectrometry. The panels show relative abundance of the indicated hemoglobin chains in reference healthy control and homozygous proband and heterozygous parents in Family 2: (A) HBB, (B) HBD, (C) HBG2, and (D) HBG1.

The intra-assay precision for quantitation of different globin chains and globin ratios was demonstrated using the samples from homozygous and heterozygous *δβ*-thalassemia along with the control samples as the reference, analyzed in triplicate. The variability in the method was expressed as CV%. The within-run CVs ranged from 0.5% to 15% for the relative abundances of different globin proteins (Table 4; Figure 6). The within-run CVs for the ***β*:*α*** ratios in the control and patient samples ranged from 2.3% to 5.2% (Table 5).

### 5.1 Molecular confirmation of LC-MS/MS results using MLPA

The results from LC-MS/MS analysis were confirmed using MLPA. The DNA acquired from proband and parents from both families were subjected to MLPA analysis. The analysis revealed homozygous deletion of the *HBB, HBD*, and *HBG1* regions in the beta globin (*HBB*) gene cluster in the proband and heterozygous deletions of this region in their parents. From these results, diagnosis of the patient likely being affected with beta gene-cluster deletion and a possible Indian inversion-deletion rearrangement (A*γδβ*)^0^ type thalassemia was made (Figure S2A,B).

## 6 Discussion

*δβ*-thalassemia is a rare autosomal recessive disorder due to large deletions in the *β*-globin gene cluster involving the *δ*- and *β*-globin genes [5, 28, 29]. As a result, the *γ*-globin genes may escape the developmental down regulation and continue to be active into adult life [30]. In some genetic conditions, such as *δβ*-thalassemia and HPFH, a high level of HbF continues into adulthood [31, 32]. There is a thin line in the clinical and hematological differences between HPFH and *δβ*-thalassemia. Thus, the level of HbF alone often cannot differentiate between the two conditions-this necessitates a molecular characterization of the defect. In HPFH, the fetal genes are overexpressed and the adult genes are underexpressed, but their combined output remains within or very close to the normal range. In contrast, patients with *δβ*-thalassemia exhibits an increased production of the *γ*-globin chains, which does not completely compensate for the decreased/absent production of the *β*-globin chains leading to high degree of globin chain imbalance [33]. Because the existing biochemical methods employed in the diagnosis of thalassemias and hemoglobinopathies are based on analysis of the tetrameric structure of hemoglobin, it is not possible to quantify individual globin chains to confirm this imbalance using these methods. We therefore used LC-MS/MS to quantitate the individual globin chains for the diagnosis of *δβ*-thalassemia. It was evident from our results that the degree of imbalance in *β*/*α* globin-chain was severe in *δβ*-thalassemia homozygous probands compared to their heterozygous parents, which was variable.

At the molecular level, these disorders can be subdivided according to the amount of HbF produced and whether it contains both ^G^*γ* and ^A^*γ* chains or only ^G^*γ* chains. Human HbF comprises two types of gamma globin chains-^G^*γ* and ^A^*γ* characterized by the presence of glycine or alanine at the position 136. Their production is controlled by two separate structural genes. The relative amounts of these two chains decrease at different rates postnatally. The ratio of ^G^*γ*:^A^*γ* is 3:1 at birth and is 2:3 in adults [34]. However, this ratio can be altered in different hemoglobinopathies as in the present study. The significant variation in ^G^*γ*:^A^*γ* ratio suggests an unequal repression of the ^G^*γ* and ^A^*γ* structural genes [35]. Nonetheless, there are no analytical methods used in current diagnosis to separate ^G^*γ* from ^A^*γ* chains. The study of gamma globin alteration in individuals is relevant from clinical point of view in that unusually high HbF levels in adult patients with various types of hemoglobinopathies may be associated with amelioration of their clinical conditions. Also, characterization of these disorders is essential in understanding the control of *γδβ* gene complex with respect to gene therapy which is being explored in thalassemias. The use of LC-MS/MS has allowed us to carry out an analysis of *γ*-globin chain production in individual patients.

The extremely high levels of ^G^*γ* in the probands in our study could be explained only by an almost complete suppression of ^A^*γ* production. The severe reduction in ^A^*γ* chain and an unusually high ^G^*γ*/^A^*γ* ratio in the probands led us to a diagnosis of homozygous ^G^*γ*(^A^*γδβ*)^0^ thalassemia. Similarly, the modest increase in ^G^*γ*/^A^*γ* ratio in parents revealed the heterozygous state in both parents of the two families. The normal HbA2 levels in both parents by HPLC and the levels of delta globin chains by direct quantitation exclude the possibility of homozygous *β*-thalassemia or double heterozygosity for *β*-thalassemia and *δβ*-thalassemia in the present cases. This was confirmed by *HBB* gene sequencing for mutation analysis of both parents in one of the families, which revealed no pathogenic mutation in the *HBB* gene.

We demonstrated the utility of the direct quantitation of globin chains and the use of *β*:*α*, ^G^*γ*:^A^*γ* globin ratios in identifying the subtype of *δβ*-thalassemia using an LC-MS/MS-based approach. The results of this study demonstrate the utility of globin protein quantitation and globin peptide ratios for the effective screening and diagnosis of *δβ*-thalassemia cases and carriers.

## 7 Conclusions

Direct quantitation of globin chains using an LC-MS/MS-based approach aids in the detection of the individual globin protein levels. This directly reflects any imbalance of globin chains and provides an accurate and rapid diagnosis of rare hemoglobinopathies such as *δβ*-thalassemia without the need for multitude of tests. The globin ratio should also assist in differentiating related variants such as HPFH as well as co-inheritance of beta thalassemia. We believe that the characterization method used in this study will prove to be useful by complementing routine hemoglobin analysis to determine the genotype and will facilitate the accurate diagnosis of (*δβ*)^0^ thalassemia and other hemoglobinopathies. To our knowledge, this is the first report of a rare variant, ^G^*γ*(^A^*γδβ*)^0^ thalassemia, validated using tandem mass spectrometry.

## Supplementary Material

Supplementary file

## Figures and Tables

**Table 1:** Summary of hematological and biochemical investigations from clinical records.

	Family 1				Family 2			
Parameter	Proband	Mother	Father		Proband	Mother	Father	
Age	10 years	30 years	35 years		8 months	26 years	30 years	
Hemoglobin	**2.9 g/dL**	**11.7 g/dL**	13.2 g/dL		**8.5 g/dL**	**11.4 g/dL**	**12.1 g/dL**	
RBCs	**1.19 × 10^6^/*μ*L**	4.12× 10^6^/*μ*L	4.15× 10^6^/*μ*L		**3.99 × 10^6^/*μ*L**	**5.96 × 10^6^/*μ*L**	5.3× 10^6^/*μ*L	
PCV	**10.5%**	37.5%	40.7%		**27.6%**	37.0%	36.4%	
MCV	88.3 fL	90.8 fL	98.2 fL		69.0 fL	62.1 fL	68.7 fL	
MCH	**24.7 pg**	28.5 pg	31.8 pg		**21.0 pg**	**19.1 pg**	22.8 pg	
MCHC	**28.0 g/dL**	31.3 g/dL	32.4 g/dL		**30.4 g/dL**	**30.8 g/dL**	32.2 g/dL	
RDW-CV	Flagged *	**19.9%**	**15.2%**		**27.7%**	**19.3%**	**19.3%**	
RDW-SD	Flagged *	**77.2 fL**	**63.3 fL**		**78.5 fL**	**50.1 fL**	53fL	
WBCs	**17.96 × 10^3^/*μ*L**	7.39× 10^3^/*μ*L	7.05× 10^3^/*μ*L		12.96 × 10^3^/*μ*L	8.3× 10^3^/*μ*L	12.7× 10^3^/*μ*L	
Platelets	152× 10^3^/*μ*L	368× 10^3^/*μ*L	240× 10^3^/*μ*L		156× 10^3^/*μ*L	348 × 10^3^/*μ*L	417× 10^3^/*μ*L	
Peripheral smear	RBC: microcytic hypochromic cells, macroovalocytes, teardrop cells, anisopoikilocytosis, NRBCs-8-10/100 WBCs, microspherocytes WBC:Many hypersegmented neutrophils Platelets: adequate	RBC: Normocytic normochromic cel Is, fragmented cells, teardrop cells, anisocytosis, macroovalocytes WBCs:Many hypersegmented neutrophils Platelets: adequate	RBC: Normocytic normochromic cel Is, fragmented cells, teardrop cells, target cel Is, macrocytes, macroovalocytes WBCs:Many hypersegmented neutrophils Platelets: adequate		RBC: microcytic hypochromic cel Is with moderate degree of anisopoikilocytosis, many target cel Is, dacrocytes, elongated cells, schistocytes, microspherocytes and macro-ovalocytes, marked increase in nRBCs – 40–48 cells/lOOWBCs WBC:Many hypersegmented neutrophils Platelets: adequate	RBC: microcytic hypochromic cel Is with erythrocytosis (suggestive of haemolytic picture) associated with megaloblastic changes WBC: hypersegmented neutrophils	RBC: microcytic hypochromic cel Is with mild erythrocytosis (suggestive of haemolytic picture) associated with megaloblastic changes WBC: neutrophilia, hypersegmented neutrophils	
Ferritin	365.8 ng/mL	12.35 ng/mL	49.34 ng/mL		133 ng/dL	Not available	Not available	
Folate	2.64 ng/mL	4.62 ng/mL	1.73 ng/mL		Not done			
Vitamin B12	<**50 pg/mL**	**79.32 pg/mL**	<**50 pg/mL**		293.0 pg/mL			
Iron profile	Not done	Not done	Not done		TIBC:455/*μ*g/dL Transferrin saturation: 29%			
**HPLC pattern**								
HbA	3.4%	80.8%	76.6%		*	89.4%	87.9%	
HbF	**96.6%**	**16.4%**	**20.6%**		**101.1%**	**8.1%**	**9.8%**	
HbA2	0%	2.8%	2.8%		*	2.5%	2.3%	
**Molecular analysis**								
MLPA analysis	Homozygous deletion of HBB, HBD and HBGl region; possibly Indian inversion-deletion rearrangement (^A^*γδβ*) type.	Heterozygous deletion of HBB, HBD and HBG1 region; possibly Indian inversion-deletion rearrangement (^A^*γδβ*) type.	Heterozygous deletion of HBB, HBDandHBGl region; possibly Indian inversion-deletion rearrangement (^A^*γδβ*) type.		Homozygous deletion of HBB to HBG1 region; possibly ^G^*γ*(^A^*γδβ*)°-thalassemia	Heterozygous deletion of HBB to HBG1 region; possibly ^G^*γ*(^A^*γδβ*)°-thalassemia	Heterozygous deletion of HBB to HBG1 region; possibly ^G^*γ*(^A^*γδβ*)°-thalassemia	
*HBB* gene sequencing for mutation analysis	-	-	-		-	No Pathogenic mutation	No Pathogenic mutation	

* Values outside of expected range.

**Table 2:** A list of selected unique peptides for the calculation of globin chain ratios.

Gene symbol	Protein/globin chain	Peptide sequence	m/z (Da)
*HBA*	Hemoglobin subunit Alpha (*α*)	VGAHAGEYGAEALER	765.37
		TYFPHFDLSHGSAQVK	917.45
		FLASVSTVLTSK	626.86
*HBB*	Hemoglobin subunit Beta (*β*)	SAVTALWGK	466.76
		VNVDEVGGEALGR	657.83
		EFTPPVQAAYQK	689.85
*HBG1*	Hemoglobin subunit Gamma-1 (^A^*γ*)	MVTAVASALSSR	596.82
*HBG2*	Hemoglobin subunit Gamma-2 (^G^*γ*)	MVTGVASALSSR	589.81

**Table 3:** Comparison of *β*/*α* and ^G^*γ*/^A^*γ* globin chain ratios in heterozygous and homozygous delta beta thalassemia.

Globin chain ratio	Family 1				Family 2					
	Proband	Mother	Father		Proband	Mother	Father	
*β/α*	0.002	0.75	0.75		0.009	1.3	1.3	
^G^ *γ* */* ^A^ *γ*	143922	95.8	28.9		3517.5	303.2	122.2	

**Table 4:** Coefficient of variation for intra-assay measurements of the relative abundance of globin chains.

	%CV				
Phenotype	HBB	HBD	HBG2	HBG1	
Healthy control	0.52	0.62	0.17	12.57	
*δβ*-thalassemia heterozygous (Father)	2.60	4.36	1.97	9.05	
*δβ*-thalassemia heterozygous (Mother)	2.09	3.87	0.59	0.37	
*δβ*-thalassemia homozygous (Proband)	11.73	10.40	15.41	13.68	

**Table 5:** Mean and CV% for intra-assay measurements of the *β*/*α* globin ratios.

Phenotype	Mean ratio	%CV
Healthy control	1.5	3.2
*δβ*-thalassemia heterozygous (Father)	1.3	5.2
*δβ*-thalassemia heterozygous (Mother)	1.3	2.3
*δβ*-thalassemia homozygous (Proband)	0.006	4.3

## Data Availability

The mass spectrometry proteomics data generated and analyzed during the current study has been deposited to the ProteomeXchange Consortium via the PRIDE partner repository with the dataset identifier PXD044775.

## Statement of significance of the study

Traditional methods for thalassemia screening by charge-sensitive methods such as capillary electrophoresis (CE) and high-performance liquid chromatography (HPLC) present inherent ambiguity due to their migration and elution patterns. These methods usually rely on the structural integrity of the hemoglobin molecule, which can be affected by the hemolysis of red blood cells and hemoglobin degradation. The confirmation requires genetic testing. However, commonly used genetic diagnostic tests target point mutations and small insertions/deletions and tend to miss large deletion mutations, thus warranting other molecular assays. Overall, the diagnosis of thalassemias involves a multitude of tests leading to delays in diagnosis. Here, we propose direct globin quantitation using tandem mass spectrometry to detect functional abnormalities of human hemoglobin such as thalassemias, providing an alternative first line screening assay.

## Abbreviations

HbF: fetal hemoglobin
Hb: hemoglobin
HBA: hemoglobin subunit alpha
HBB: hemoglobin subunit beta
HBD: hemoglobin subunit delta
HBG1: hemoglobin subunit gamma-1
HBG2: hemoglobin subunit gamma-2
HPFH: hereditary persistence of fetal hemoglobin
LCR: locus control region
HbA/HbA2: adult hemoglobin
MLPA: multiplex ligation-dependent probe amplification
PCR: polymerase chain reaction
LC-MS/MS: liquid chromatography with tandem mass spectrometry
CE-HPLC: cation-exchange high performance liquid chromatography
BCA: bicinchonic acid
mM: millimolar
ms: millisecond
ppm: parts per million
FDR: false discovery rate
PD: Proteome Discoverer
LFQ: label-free quantitation
PSM: peptide-spectrum match
CV: coefficient of variation
AGC: automatic gain control
HCD: higher-energy collisional dissociation
EDTA: ethylenediamine tetraacetic acid
MCV: mean corpuscular volume
MCH: mean corpuscular hemoglobin
MCHC: mean corpuscular hemoglobin concentration
RDW: red cell distribution width.
