# Supplementary material for: Identification of a rare [Gγ(Aγδβ)0] -thalassemia using tandem mass spectrometry
Source: Proteomics. Author manuscript; Available in PMC 2024 Jul 16. (PMC7616216; doi:10.1002/pmic.202300495)

SUPPORTING INFORMATION

Supplementary Figure 1: HPLC chromatogram showing elevation of HbF levels in probands. HPLC chromatograms of Proband 1 (A) and Proband 2 (B) from Family 1 and Family 2, respectively, are shown indicating HbF values outside of expected range with reduced HbA2 levels.

Supplementary Figure 2: MLPA Pattern in the probands showing deletion in the β-globin (HBB) gene cluster. The figure depicts homozygous deletion of genes in the beta globin gene cluster in Proband 1 (A) and Proband 2 (B). The deleted regions include HBB intron 2 to HBD exon 3 and HBG1 exon 3 gene regions.

Supplementary Figure 1

A

F Concentration =   96.6* %
A2 Concentration =   %

*Values outside of expected ranges

Analysis comments:

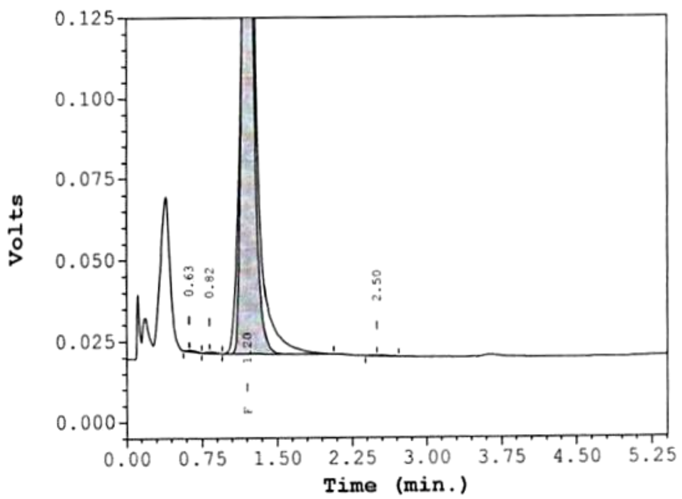

B

F Concentration =   101.1* %
A2 Concentration =   %

*Values outside of expected ranges

Analysis comments:

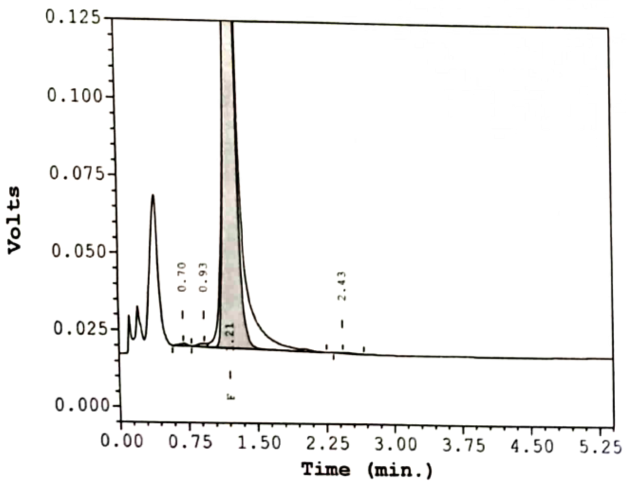

Supplementary Figure 2

A

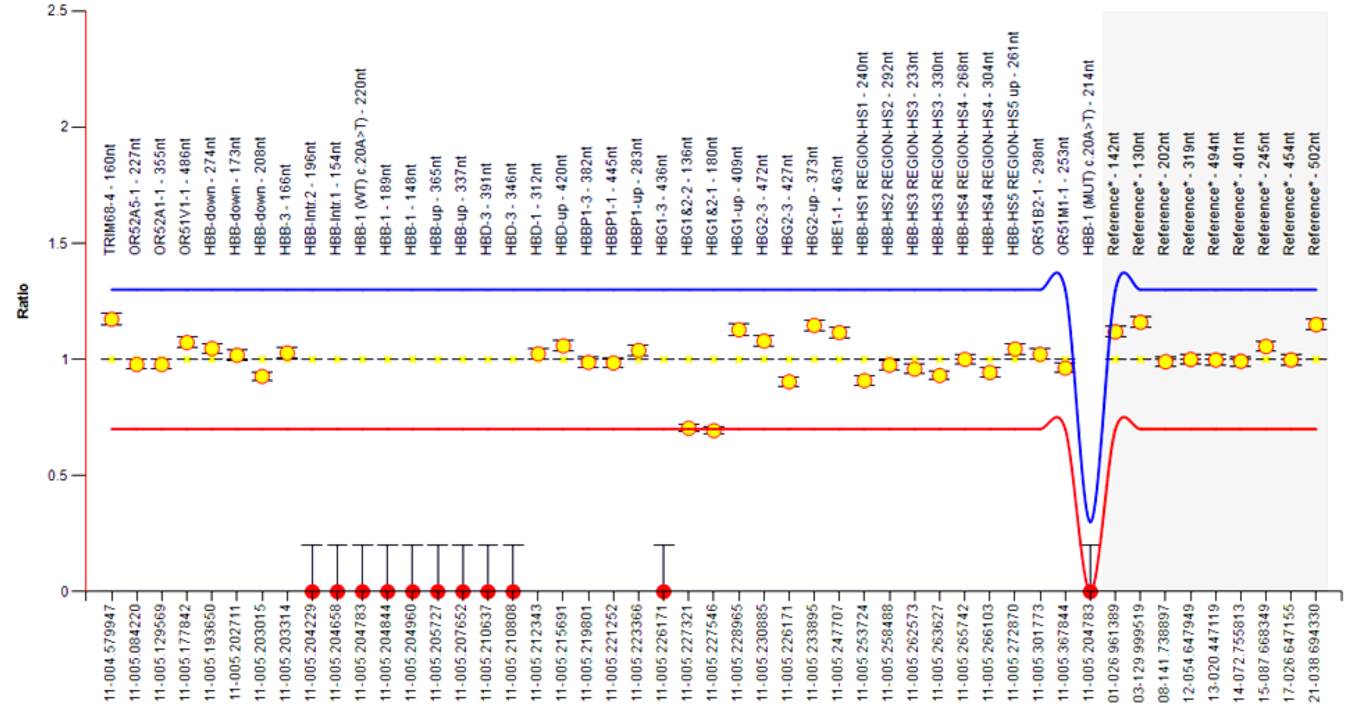

B

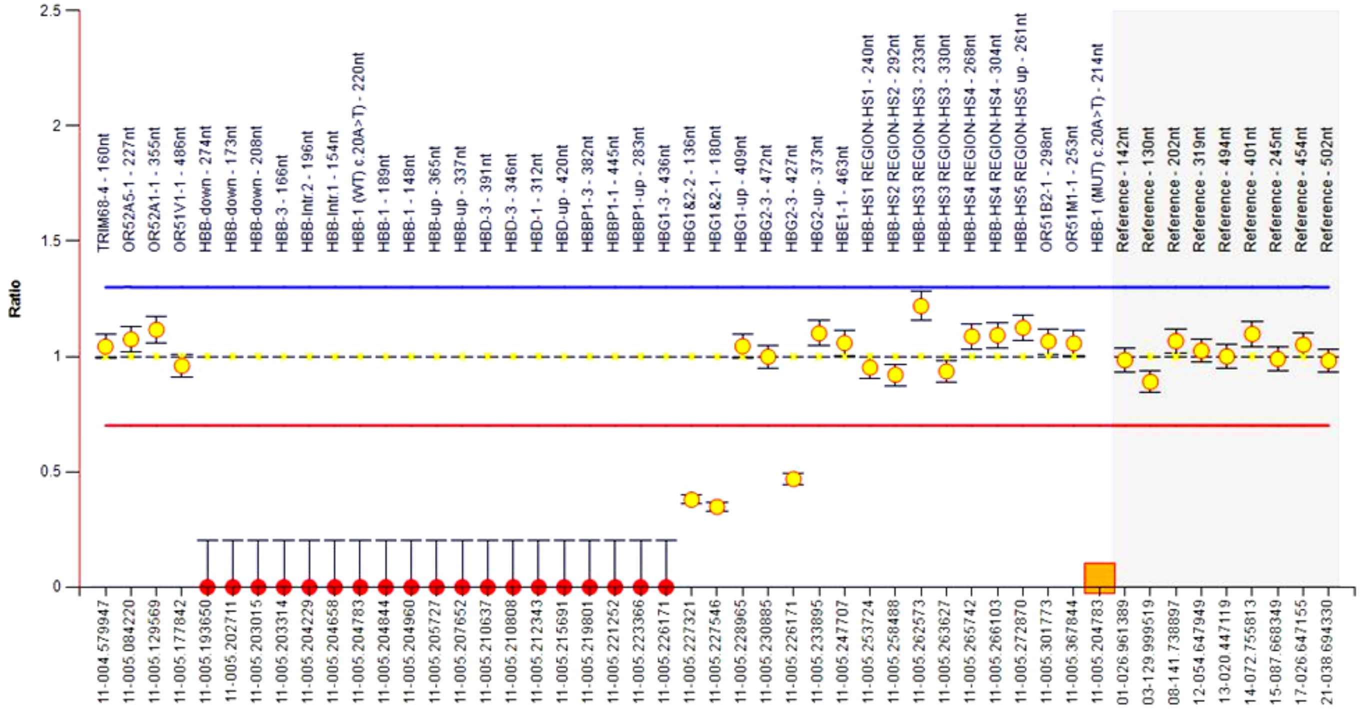

Supplement: Supplementary file [file EMS195229-supplement-Supplementary_file.pdf]
